# Sera of patients with axial spondyloarthritis (axSpA) enhance osteoclastogenic potential of monocytes isolated from healthy individuals

**DOI:** 10.1186/s12891-018-2356-4

**Published:** 2018-12-06

**Authors:** Mariusz Korkosz, Marcin Czepiel, Zofia Guła, Małgorzata Stec, Kazimierz Węglarczyk, Magdalena Rutkowska-Zapała, Anna Gruca, Marzena Lenart, Jarosław Baran, Jerzy Gąsowski, Przemysław Błyszczuk, Maciej Siedlar

**Affiliations:** 1 Department of Rheumatology, Jagiellonian University Medical College, 10 Sniadeckich Str., Krakow, Poland; 2 Department of Clinical Immunology, Institute of Paediatrics, Jagiellonian University Medical College, 265 Wielicka Str., 30-663 Krakow, Poland; 3 Department of Internal Medicine and Gerontology, Jagiellonian University Medical College, 10 Sniadeckich Str., Krakow, Poland

**Keywords:** Osteoclastogenesis, RANKL, RANK, Monocytes, Axial spondyloarthritis

## Abstract

**Background:**

Axial spondyloarthritis (axSpA) is characterized by significant bone loss caused by dysregulation of physiological bone turnover, possibly resulting from intensified differentiation of osteoclasts. The aim of this study was to reevaluate the levels of osteoclastogenesis-mediating factors: soluble RANKL, M-CSF, OPG and other cytokines in sera of untreated, with sDMARDs and/or bDMARDs, axSpA patients and to test whether these sera influence differentiation of healthy monocytes towards osteoclast lineage.

**Methods:**

Bone remodeling molecules (RANKL, M-CSF, OPG, IL-6, OSM, IL-17A, TGFβ, and TNFα) were evaluated in 27 patients with axSpA and 23 age and sex-matched controls. Disease activity (BASDAI, ASDAS) and inflammatory markers (ESR, CRP) were assessed. Monocytes obtained from healthy individuals were cultured in vitro in presence of sera from 11 randomly chosen axSpA patients and 10 controls, with addition of exogenous M-CSF and/or RANKL or without. Osteoclastic differentiation was assessed analyzing osteoclast markers (cathepsin K and RANK at mRNA level) and with osteoclast-specific staining.

**Results:**

axSpA patients’ sera levels of soluble RANKL were significantly lower and M-CSF, IL-6, OSM, IL-17A and TNFα significantly higher in comparison to controls, whereas of OPG and TGFβ were comparable in both groups. Numbers of generated in vitro osteoclasts and cathepsin K mRNA levels did not differ between cultures supplemented with sera of healthy and axSpA patients, both in the absence and presence of M-CSF. Instead, addition of exogenous RANKL boosted osteoclastogenesis, which was significantly higher in cultures with axSpA sera. Furthermore, sera from axSpA patients induced substantially higher levels of RANK mRNA, independently of M-CSF and RANKL stimulation.

**Conclusion:**

We show that, paradoxically, serum levels of soluble RANKL observed in axSpA are in fact significantly lower in comparison to healthy blood donors. Our results indicate that sera of axSpA patients - in contrary to healthy subjects - contain circulating, soluble factors (presumably IL-6, OSM, IL-17A, TNFα and others) able to stimulate healthy monocytes responsiveness to even relative low RANKL serum levels, by inducing high RANK mRNA expression and - as a net effect - boosting their osteoclastogenic potential. We suggest also that locally produced RANKL in axSpA may induce overactive osteoclasts from their precursors.

## Key messages:

1. In comparison to healthy subjects, lower serum level of free soluble RANKL is observed in axSpA patients.
2. Sera from axSpA patients influence in vitro osteoclastogenesis by up-regulation of RANK mRNA in healthy monocytes.

## Background

Human receptor activator of nuclear factor–κB ligand (RANKL) and monocyte colony-stimulating factor (M-CSF) are two key molecules for osteoclast formation from peripheral blood monocytes and their activation, whereas osteoprotegerin (OPG) acts as a decoy receptor for the RANKL [1]. These cytokines play critical role in physiological bone turnover and their dysregulation leads to impairment of osteoclasts generation and excessive bone formation, as observed in numerous rheumatic diseases including spondyloarthritis (SpA). Abnormal serum levels of M-CSF and RANKL may thus represent a crucial factor in SpA pathology. There is however substantial discrepancy regarding serum RANKL levels in SpA patients (especially axSpA). Previous studies reported either higher or unchanged serum RANKL levels in axial spondyloarthritis (axSpA) patients in comparison to healthy subjects [2–5]. Therefore, in this study we especially aimed to reevaluate the levels of key osteoclastogenesis mediators – soluble RANKL and M-CSF - in sera of axSpA patients.

Osteoclastogenesis is also induced by several other cytokines, including tumor necrosis factor alpha (TNFα), Interleukin 6 (IL-6), IL-17A, or transforming growth factor β (TGFβ) [6–9]. The role of RANKL-independent (non-canonical) pathways of osteoclast formation and activation seems to be especially important in inflammatory rheumatic diseases such as axSpA, rather than in physiological bone resorption or postmenopausal osteoporosis [6]. Moreover, as demonstrated recently, in ankylosing spondylitis (AS), osteoclast precursors (monocytes) show impaired osteoclasts characteristic gene expression profile [5]. It is unclear, whether this is a result of aberrant extracellular stimulation of monocytes circulating in axSpA patients’ body fluids, their inherited trait, or both.

Considering the potential important role of non-canonical osteoclastogenesis in SpA pathophysiology we evaluated levels of several additional cytokines (namely IL-6, Oncostatin M (OSM), IL-17A, TGFβ, and TNFα) in sera of studied groups. Consequently, by utilising in-vitro differentiation system, we examined whether these sera (being a natural milieu of monocytes) influence healthy monocyte differentiation towards osteoclasts, to avoid potential interference with inherited disregulation of physiological monocyte functions in axSpA patients. For that, in serum-driven differentiated cells we determined the gene expression of osteoclast markers such as cathepsin K and RANK as well as performed osteoclast-specific cell staining. Our results indicate that RANKL-independent, serum soluble factors-mediated osteoclastogenesis may indeed play a significant role in axSpA bone remodeling by substantially influencing expression of RANK mRNA in healthy osteoclast precursors.

## Patients and methods

### Patients and controls

Twenty-seven patients with axSpA (18 AS, 9 nr-axSpA) according to the Assessment of SpondyloArthritis International Society (ASAS) classification criteria and 23 healthy age and sex-matched controls were enrolled in this study [10]. Patients were under 45 years, with relatively recent disease onset (not exceeding 10 years of symptoms duration), naïve to synthetic and/or biological Disease Modifying Anti-Rheumatic Drugs (sDMARDs, bDMARDs) and without the administration of systemic glucocorticosteroids. Patients provided a signed informed consent and the study protocol was approved by the local bioethics committee.

### ELISA and Cytometric bead Array (CBA) of bone remodeling molecules

Serum for bone remodeling molecules was obtained from axSpA patients and controls and stored at − 20 °C. RANKL, oncostatin M (OSM) (Elabscience Biotechnology Co. Ltd., Wuhan, China), (Abbexa, Cambridge, UK), M-CSF (R&D Systems, Minneapolis, MN, USA), OPG (Ray-Biotech, Norcross GA, USA) and TGFβ (Thermo Fisher Scientific Waltham, MA USA) were assessed by ELISA. We used the RANKL kit which according to the manufacturer has been developed from the human Tumor Necrosis Factor ligand superfamily member 11 protein where the sequence of the immunogen rests within the region Ile140~Asp317 and hence this kit is designed to detect free soluble RANKL. The presented M-CSF levels were assayed in a subsample of 13 patients due to limited availability of patients’ material. Samples were run in duplicates, according to the manufacturers protocols, and results were obtained using an ELISA reader (BioTek Instruments, Vinooski, VT).

Concentrations of IL-6, IL-17A, TNFα in the serum were measured using the CBA system (BD Biosciences) followed by flow cytometric analysis (FACS Canto). The CBA beads were discriminated in FL-4 and FL-5 channels, while the concentration of specified cytokines was determined by the intensity of FL-2 fluorescence, using the respective standard reference curve and FCAP Array software (BD Biosciences).

The detection levels were < 58 pg/ml for RANKL, 11 pg/ml for M-CSF and 1 pg/ml for OPG, 9,38 pg/ml for OSM, 156,3 pg/ml for TGFβ, 2,4 pg/ml for IL-6, 18,9 pg/ml for IL-17A and 3,8 pg/ml for TNFα.

### Cell culture of osteoclasts for cathepsin K and RANK mRNA expression analysis

- Differentiation of monocytes into osteoclasts

Peripheral blood from healthy donors was used to isolate monocytes, as described previously [11]. Briefly, peripheral blood mononuclear cells (PBMC) were isolated by the standard Ficoll/Isopaque (Pharmacia, Uppsala, Sweden) density gradient centrifugation. Subsequent separation of monocytes form PBMC was performed with JE-5.0 elutriation system, equipped with the Sanderson separation chamber (Beckman, Palo Alto, CA, USA) [11]. Isolation purity was over 95% as tested by staining with anti-CD14 mAb (BD Biosciences Pharmingen, San Diego, CA) and flow cytometry analysis (FACSCanto flow cytometer, Becton Dickinson, San Jose, CA, USA). Monocytes were seeded in 24-well plates at density of 2 × 10^5^ cell/cm^2^ and cultured in RPMI 1640 medium (Corning) supplemented with 10% serum (from randomly chosen axial SpA patients, *n* = 11, or heathy controls, *n* = 10) and 50 μg/ml gentamycin for 14 days (with half of media change every other day). Cells were cultured with rhM-CSF (10 ng/ml) alone, or rhM-CSF and rhRANKL (10 ng/ml) (both from Peprotech, London, UK), or without addition of additional factors for the whole differentiation period. After 14 days cells were harvested for RNA isolation (Norgen, RNA purification kit).

b. Analysis of qRT-PCR

RNA (200 ng) from cultured cells was transcribed using NG dART RT kit (EurX, Poland). qPCR for studied genes was performed using SG qPCR Master Mix (EurX, Poland) with QuantStudio 7 cycler (Thermo Scientific). The CT values were normalised to housekeeping gene – GAPDH. Results were analysed using standard 2^-^^ΔΔCT^ method. Primer sequences used in this study: GAPDH_FW: AGATCATCAGCAATGCCTCCT, GAPDH_RV: TGGTCATGAGTCCTTCCACG, Cathepsin K_FW: TTCCCGCAGTAATGACACCC, Cathepsin K_RV: GGAACCACACTGACCCTGAT, RANK_FW: TGGGACGGTGCTGTAACAAA, RANK_RV: CCAAGTATTCATCCGGGCCA.

### Tartrate-resistant acid phosphate (TRAP) activity detection in monocyte-derived osteoclasts

For TRAP activity assay, monocytes were isolated and cultured as described above for 20 days (with half of cell culture medium refreshed every 3–4 days). Cells were then fixed and stained for TRAP activity using commercially available kit according to manufacturer’s protocol (Sigma-Aldrich, Acid Phosphatase, Leukocyte [TRAP] Kit). Large multinucleated cells (containing at least 3 nuclei) showing TRAP activity were considered as osteoclasts. For quantification of osteoclast differentiation efficiency, cells fulfilling osteoclast criteria (TRAP activity + at least 3 nuclei) were counted (*n* = 12 representative microscope images for each condition) and the percentage of osteoclasts was calculated.

### X-ray of sacroiliac joints, cervical and lumbar spine

To assess structural damage radiographs of sacroiliac joints were analyzed according to modified New York (mNY) criteria; radiographs of the lateral cervical and lumbar spine were collected to calculate the mSASSS (modified Stoke Ankylosing Spondylitis Spinal Score).

### Statistical analysis

Database management and analysis were performed using SAS 9.2 (SAS Institute Inc. Cary, NC, USA) and GraphPad PRISM (San Diego, CA) software packages. The variables following a non-normal distribution are presented as medians (25th–75th percentile) and those that are normally distributed as means ± standard deviations (SD). The non-normally distributed data were analyzed using nonparametric techniques (Wilcoxon’s test for comparison of unpaired continuous data). The means of normally distributed variables were compared using Student’s *t*-test. The proportions were compared using chi-square test. All *P*values were two-tailed and 5% was considered as the threshold for significance.

## Results

### Demographic, clinical, inflammatory markers and x-ray data

Table 1 presents characteristics of patients and controls. Mean age (years, SD) of studied patients was 32.9 (7.7), the mean disease duration (years, p25-p75) was 7.5 (5–10) and 59.5% were male. A total of 66.7% of axSpA patients fulfilled the mNY x-ray criteria for ankylosing spondylitis (AS).

**Table 1:** Characteristic of axSpA patients and healthy blood donor groups

	axSpA patients	Healthy subjects	*P*value
n, (%)	27 (56.25)	23 (43.75)	
Age, mean ± SD years	32.9 (7.7)	35.1 (5.4)	0.31
Sex, % male	59.5	52.4	0.61
HLA B27 positive, %	91.7	N/A	
Duration of symptoms, median (p25-p75) years	7.5 (5–10)	N/A	
IBP, %	88.5	N/A	
CRP, median (p25-p75) mg/l	8.8 (2.4–12.9)	0.27 (0.18–0.54)^a)^	< 0.0001
ESR, median (p25-p75) mm/h	22.5 (15.0–31.0)	N/A	
BASDAI (0–10 scale), median (p25-p75)	2.1 (0.8–4.4)	N/A	
ASDAS-CRP median (p25-p75)	2.1 (1.6–3.1)	N/A	
mNY x-ray criteria for AS (%)	66.7	N/A	
mSASSS median (p25-p75)	3 (0–72)	N/A	

*IBP* inflammatory back pain, *CRP* C-reactive protein, *ESR* erythrocyte sedimentation rate, *BASDAI* Bath Ankylosing Spondylitis Disease Activity Index, *ASDAS* Ankylosing Spondylitis Disease Activity Score, *mNY* modified New York, *mSASSS* modified Stokes Ankylosing Spondylitis Spinal Score

^a)^data obtained from randomly chosen 9 healthy blood donors

### Concentration of canonical osteoclastogenesis promoting molecules in sera of axSpA patients and healthy donors

**Fig. 1:**
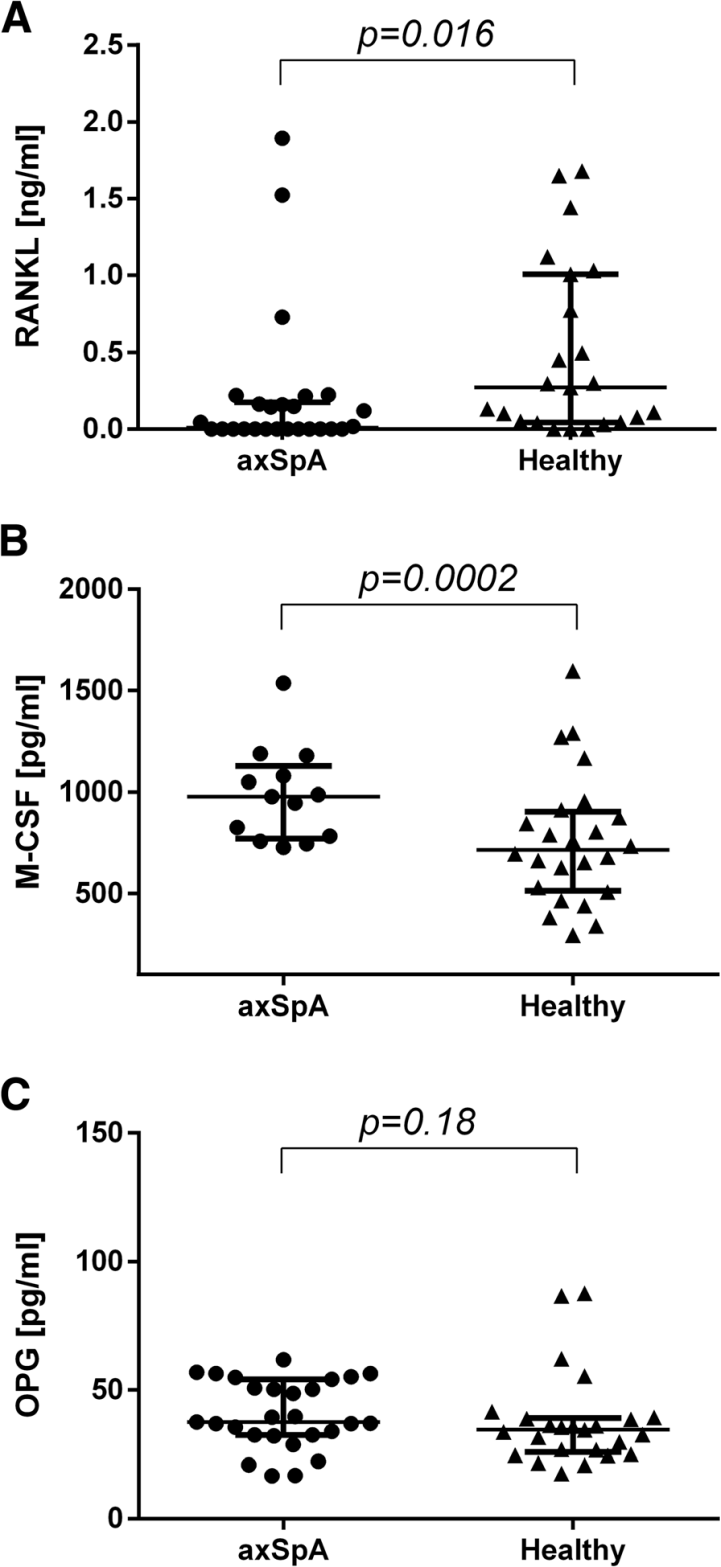
Determination of RANKL, M-CSF and OPG serum levels. Concentrations of RANKL, M-CSF and OPG in sera of axSpA patients (*n* = 27) and healthy individuals (*n* = 23). RANKL was found significantly lower (*P* = 0.016; median values: 8.5 vs. 272 pg/ml) (**a**) and M-CSF significantly higher (*P* = 0.0002; median values: 978.2 vs. 715.5 pg/ml) (**b**) in axSpA patients’ sera. OPG levels were comparable (*P* = 0.18) (**c**). Data presentation: Median + interquartile ranges. Statistics: Wilcoxon’s test for non-normally distributed data

Concentrations of main osteoclastogenesis molecules, i.e. RANKL and M-CSF were determined in sera from axSpA patients and healthy donors. Sera levels of RANKL in axSpA patients appeared significantly lower (*P* = 0.016; median values: 8.5 vs. 272 pg/ml) and M-CSF significantly higher (*P* = 0.0002; median values: 978.2 vs. 715.5 pg/ml) in comparison to healthy subjects (Fig. 1a, b). OPG levels in axSpA did not differ from those in healthy blood donors (*P* = 0.18) (Fig. 1c). Concentrations of these factors were comparable upon division of the group of axSpA patients into subgroups of non-radiographic SpA and AS (data not shown).

### Cell culture of osteoclasts for mRNA expression of Cathepsin K and RANK

**Fig. 2:**
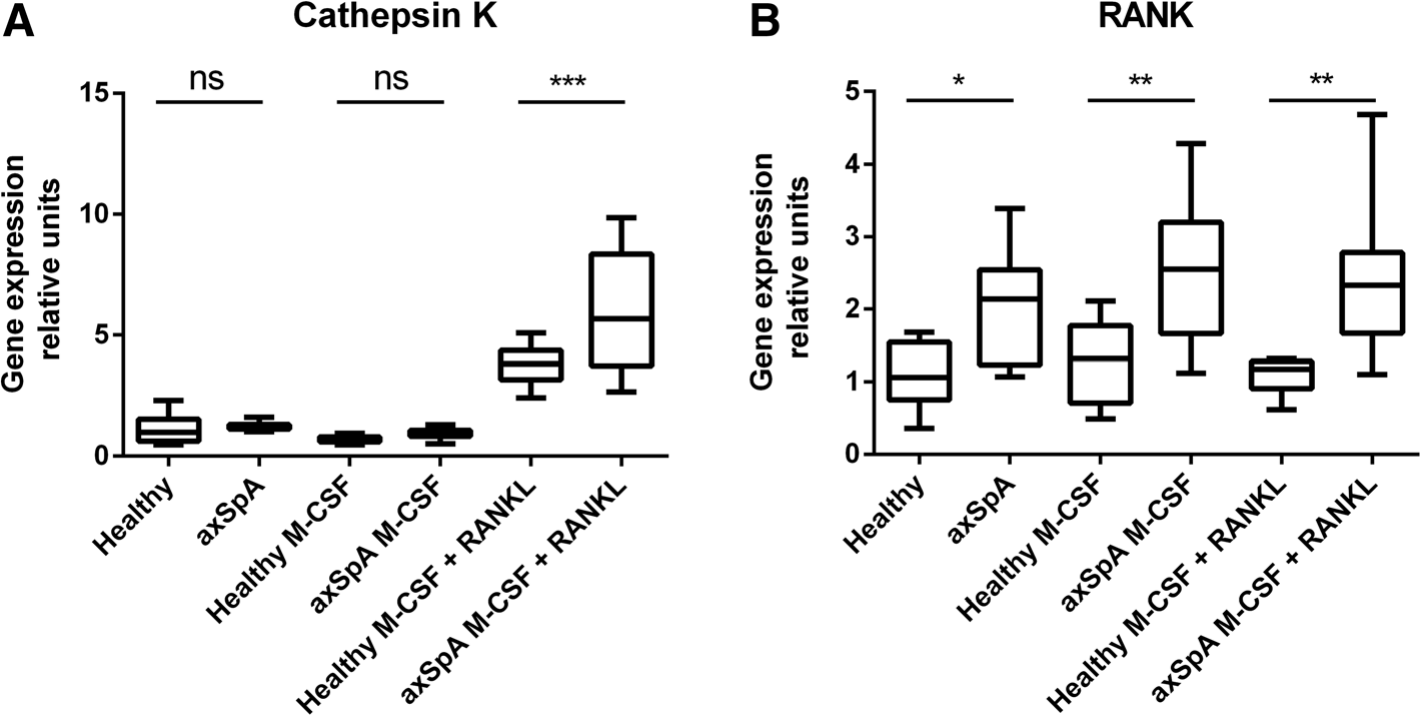
Cathepsin K and RANK gene expression analysis. qPCR analysis of Cathepsin K mRNA (**a**) and RANK mRNA (**b**) expression in monocytes differentiated to osteoclasts for 14 days in presence of serum from axSpA patients (*n* = 11) and healthy donors (*n* = 10). Addition of the exogenous rhRANKL boosted, independently of M-CSF, osteoclastogenesis which was significantly higher in cultures with axSpA sera (in comparison the control sera), as assessed by Cathepsin K mRNA expression (**a**) (*** *P* = 0.0004, mean values: 3.752 vs. 5.957). Supplementation of the culture media with rhM-CSF and/or rhRANKL did not influenced RANK mRNA expression, which was dependent only of axSpA patients sera admixture. axSpA or Healthy – sera obtained from axSpA patients or control blood donors (**b**). * (*P* = 0.0491, mean values: 1.096 vs. 2.028; ** *P* = 0.0018, mean values: 1.258 vs. 2.545; ** *P* = 0.0017, mean values: 1.081 vs. 2.378 respectively). Data presentation: box = median with interquartile ranges, whiskers – min/max value. Statistics: One-way ANOVA with Tukey’s correction for multiple testing

To determine the effect of serum factors on osteoclast differentiation, monocytes isolated from healthy donors were cultured in the presence of serum from either axSpA patients or healthy blood donors for 14 days. Expectedly, osteoclast differentiation was significantly higher when cells were cultured in medium containing ostoclastogenesis stimulants – M-CSF and RANKL, as revealed by qRT-PCR analysis for Cathepsin K – marker of mature osteoclasts. Intriguingly, Cathepsin K mRNA expression was stimulated significantly stronger in presence of serum from axSpA patients compared to the corresponding healthy group (Fig. 2a). In order to elucidate the possible cause of the observed difference we decided to measure the level of RANK transcripts in these cells, that would indicate the relative abundance of RANKL receptor at the cell surface. Surprisingly, RANK mRNA expression was significantly higher in all tested culture conditions in which serum from axSpA patients was used compared to the corresponding healthy serum groups. Importantly, induction of RANK mRNA expression by axSpA serum was independent of supplementation of cell culture medium with such factors as M-CSF or M-CSF + RANKL, as the observed stimulatory effect in all the “axSpA” conditions was comparable (i.e. not statistically different) (Fig. 2b). The above results were further corroborated using monocytes isolated from another healthy blood donor and randomly chosen sera from 6 healthy subjects and 7 axSpA patients (data not shown). This data indicate that serum from axSpA patients contains additional factors capable of promoting RANK mRNA expression, independently of serum RANKL and M-CSF levels.

### Tartrate-resistant acid phosphate (TRAP) activity detection in monocyte-derived osteoclasts

**Fig. 3:**
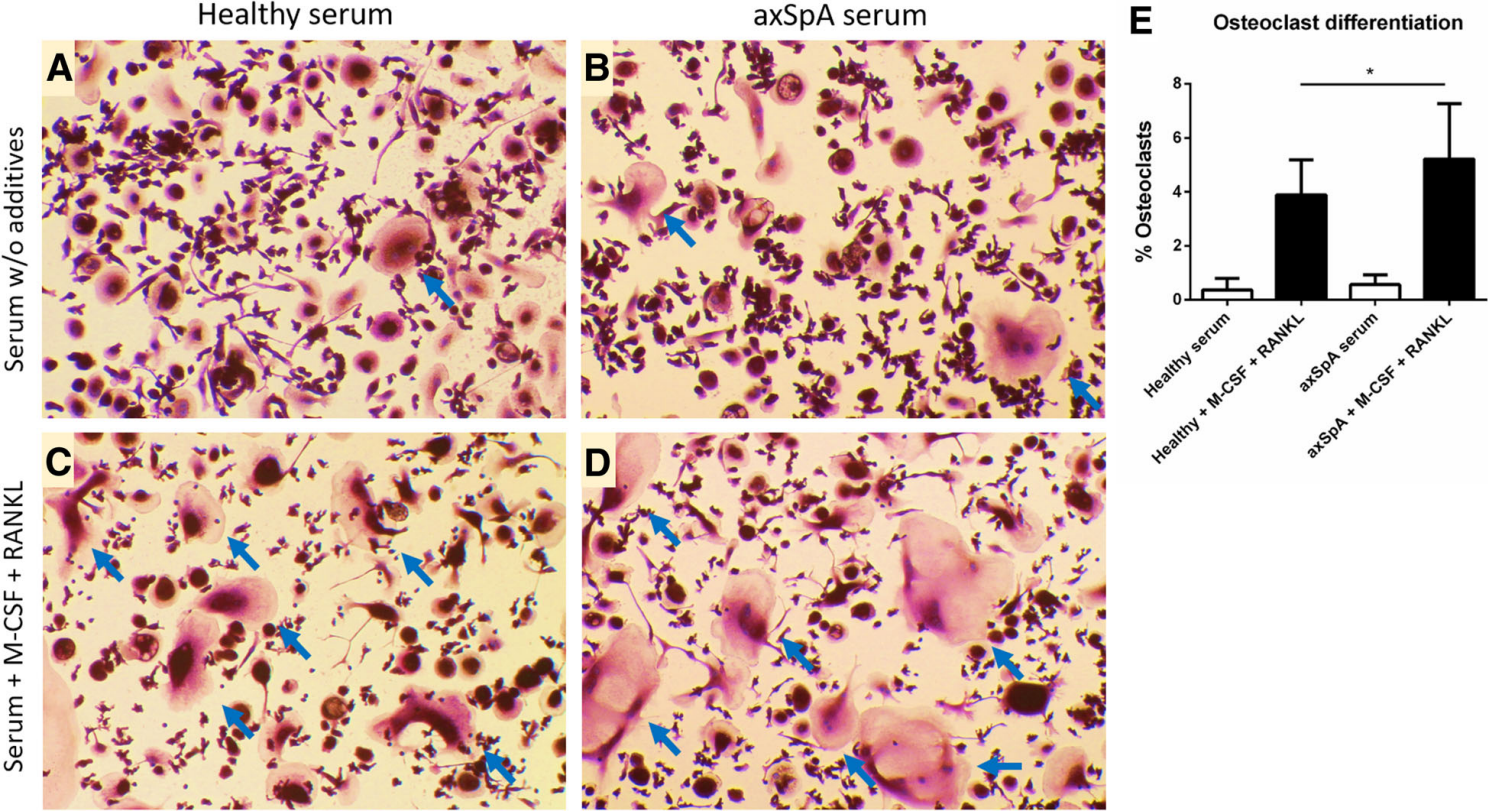
TRAP staining of monocyte-derived osteoclasts. Monocytes were differentiated to osteoclasts for 20 days in presence of serum from axSpA patients and healthy donors as described in “materials & methods”. **a**-**d** Cells were fixed and stained to detect TRAP activity. TRAP-positive (violet-labeled) cells with at least three nuclei were considered osteoclasts (blue arrows). **e** Quantification of osteoclast differentiation efficiency. Graph represent the mean + SD quantified form *n* = 12 representative micrographs for each condition. (*P = 0.0344; mean values: 3.9 vs. 5.2%). Statistics: One-way ANOVA with Tukey’s correction for multiple testing

To confirm that isolated monocytes differentiate into osteoclasts in response to human sera, we subjected the 20 day in vitro monocyte cultures to TRAP activity detection (an well-established osteoclasts marker). We noticed that monocytes cultured in presence of both axSpA or healthy sera, but without supplementation with exogenous factors, only sporadically differentiated into osteoclasts – large, TRAP-positive cells with at least three nuclei (Fig. 3a, b). On the other hand, addition of M-CSF and RANKL to the cell culture medium greatly increased osteoclast differentiation efficiency (Fig. 3c, d). Yet, although cells with osteoclast phenotype were identified in cultures using axSpA as well as healthy serum, osteoclasts differentiation was more efficient in presence of axSpA serum (Fig. 3e; 5.2 vs. 3.9%) and the derived cells were bigger and more mature (Fig. 3d), which seems to correspond well to the qRT-PCR results presented above.

### Concentration of non-canonical (i.e. RANKL-independent) osteoclastogenesis factors in studied sera

**Fig. 4:**
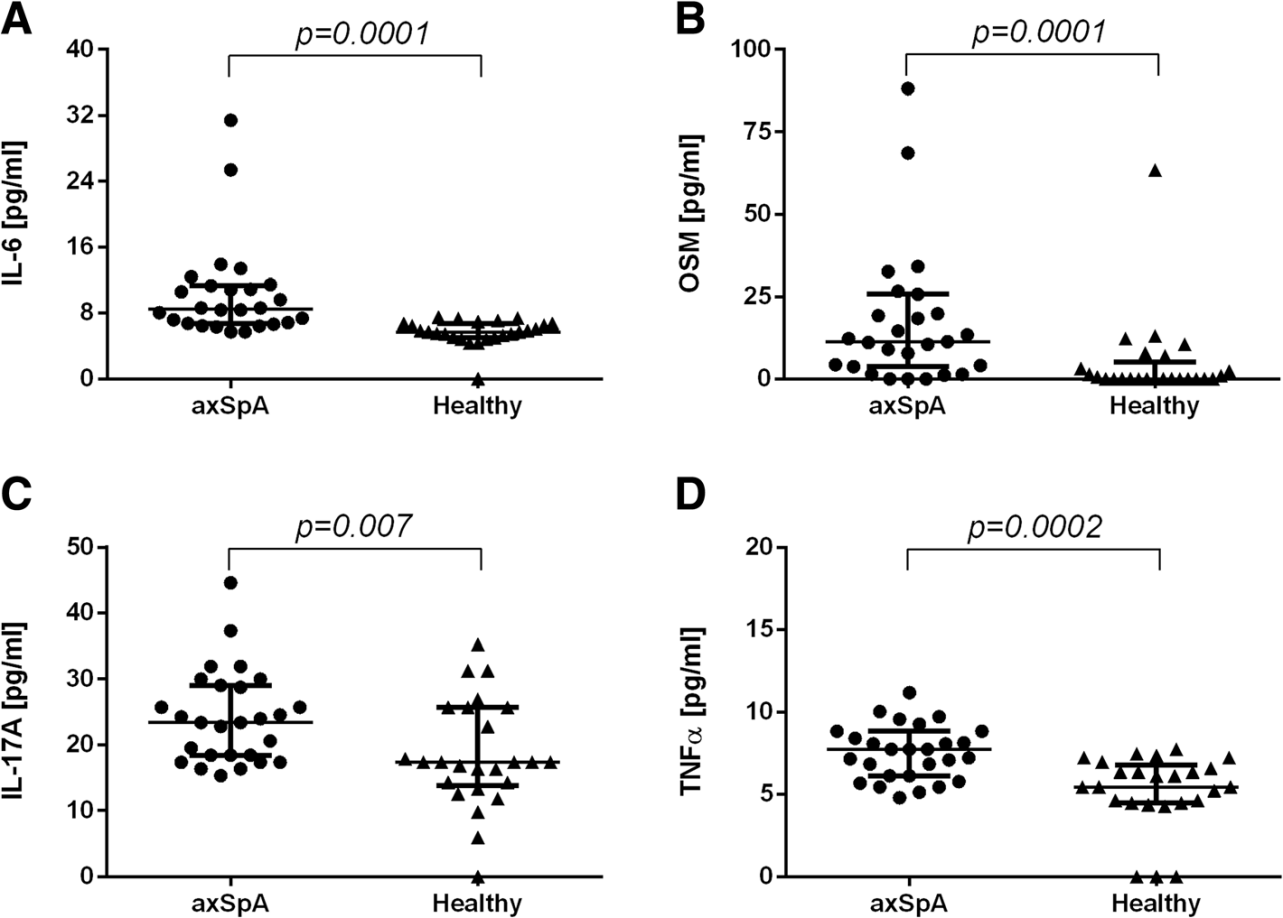
Serum levels of non-canonical osteoclastogenesis-promoting factors. Concentrations of IL-6, OSM and IL-17A and TNFα (**a**-**d**) in sera of axSpA patients (*n* = 27) and healthy individuals (*n* = 23). Levels of examined cytokines were found significantly higher in axSpA patients’ sera (median values: 8.515 vs. 5.710; 11.39 vs. 0.0; 23.39 vs. 17.37; 7.740 vs. 5.450 pg/ml respectively). Data presentation: Median with interquartile ranges. Statistics: Wilcoxon’s test for non-normally distributed data

In vitro osteoclast differentiation data indicates that axSpA patients’ sera contains factors other than RANKL and M-CSF that are capable of driving osteoclast generation from monocytes. In search of possible candidates, sera levels of so-called non-canonical osteoclastogenesis factors such as IL-6, OSM, IL-17A, TNFα and TGFβ were determined. Notably, levels of IL-6, OSM, IL-17A and TNFα were significantly elevated in axSpA patients’ sera compared to sera of healthy donors (Fig. 4a-d; median values: 8.515 vs. 5.710; 11.39 vs. 0.0; 23.39 vs. 17.37; 7.740 vs. 5.450 pg/ml respectively). Concentrations of TGFβ did not differ between studied groups (data not shown). IL-6, OSM, IL-17A and TNFα have been shown to promote osteoclastogenesis in both RANKL-dependent and RANKL-independent manner [6, 9, 12, 13] and thus, elevated levels of these factors in sera of axSpA patients may, at least partially, explain increased osteoclastogenesis-promoting potential for of axSpA serum as shown by in vitro differentiation experiments.

## Discussion

In this study we analyzed serum bone remodeling molecules in axSpA patients and healthy individuals along with an in vitro model of differentiation of isolated, healthy peripheral blood monocytes into active osteoclasts in various culture conditions. While a recent study by Perpétuo et al. analyzed osteoclastogenic potential of monocytes obtained from SpA patients, we evaluated for the first time the effect of axSpA patients sera on monocytes from healthy individuals, differentiated into osteoclast lineage [5].

We observed a significantly lower serum level of RANKL and higher level of M-CSF in axSpA patients. Importantly, lower RANKL concentration in sera of axSpA patients were not due to increased level of RANKL decoy receptor - OPG. Previous studies reported higher or similar levels of RANKL in sera of SpA patients compared do healthy donors [2–5]. The discrepancies in RANKL level might be due to distinctive patient’s characteristics, technical RANKL evaluation and treatment modalities [2–5]. Therefore, a rigorous selection of the SpA patients group (i.e. including only axSpA patients with relatively recent disease onset that were naïve to sDMARDs, bDMARDs and steroid treatment, as such therapies are known to influence osteoclastogenesis) should be considered as substantial advantage of the current study [14]. Another one was the use of RANKL ELISA kit designed to detect free soluble RANKL exclusively (see Methods).

In order to verify whether lower RANKL levels in sera of axSpA patients were functionally meaningful, we applied an in vitro cell culture model. We used peripheral blood monocytes isolated from healthy individuals and subjected them to differentiation towards osteoclasts using sera from axSpA patients and healthy blood donors (with osteoclastogenic inducers such as M-CSF or/and RANKL or without). We found that under control conditions (culture medium with serum alone) or in the presence of M-CSF, osteoclast activity reflected by expression of cathepsin K mRNA, was comparable for treatments with serum from axSpA patients and healthy donors. However, in the presence of exogenous RANKL (but not M-CSF), which represents key osteogenic factor, the osteoclasts activity was higher in cultures treated with sera from axSpA group. It seems that factors present in sera from controls or axSpA patients induce comparable osteoclasts generation despite of relative low RANKL levels in axSpA group. However, our data point to other factors in patient serum, which predispose monocytes for boosted osteoclastogenesis in the presence of high RANKL levels, as mimicked in our settings by stimulation with rhRANKL.

Considering lower serum RANKL in axSpA group we argue that above results may support the hypothesis that regardless of endogenous level of RANKL in axSpA, the patient sera activate healthy monocytes to accelerate osteoclast differentiation, while under exposure to RANKL. The possible mechanism is that those serum factors induce expression of RANK on monocytes as RANKL-RANK interaction is considered critical for efficient transition from monocytes to osteoclasts. We tested such a possibility and found that serum from axSpA patients – in comparison to serum from healthy donors – significantly increased the expression of RANK mRNA in monocytes. However, in this case, it was independent on exogenous M-CSF and/or RANKL. Such a result may explain why rhRANKL increased Cathepsin K mRNA expression in monocyte-derived osteoclasts in axSpA patient group. It is likely that soluble factors present in axSpA sera sensitize monocytes to osteoclastogenic response, which is in accordance with former observations (without explanation of its biological significance) of high RANK protein expression on SpA monocytes [5]. Such a preconditioning mechanism could possibly be utilised by osteoclast precursors, which, subjected to high RANKL levels within local tissue microenvironment, could efficiently and rapidly differentiate into mature osteoclasts.

In search for factors that could drive such a sensitization of osteoclasts precursors to RANKL we assessed the sera levels of several RANKL-independent osteoclastogenesis factors in axSpA patients and healthy donor groups. Although not formally proven in our work, we might assume that significantly elevated levels of IL-6, OSM, IL-17A and TNFα in axSpA sera were responsible for enhanced osteoclastogenesis, as these molecules have been already identified to be involved in non-canonical pathways of osteoclastogenesis [6, 9, 12]. TNFα, for instance, is capable of inducing ostoclastogenesis in both RANKL-independent and RANKL-synergistic way [15]. Interestingly, as shown by another group, TNFα alone failed to induce osteoclast differentiation from macrophages [16]. On the other hand, in combination with low concentrations of RANKL (100 times lower than normally required for osteoclast differentiation) TNFα potently stimulated osteoclastogenesis [16]. Given lower concentrations of RANKL and higher levels of TNFα in serum of axSpA patients observed in our study, similar bone destruction mechanisms may operate in SpA pathology. Well established is also RANKL-independent osteoclastogenesis potential of IL-6. Among others, IL-6 (in combination with M-CSF) was shown to potently stimulate osteoclast differentiation of human monocytes [17]. IL-17A, on the contrary, was clearly demonstrated to enhance osteoclastogenesis by inducing expression of RANK on human CD14-positive osteoclast precursors [18]. OSM, on the other hand, was linked to increased osteoclastogenesis when acting through OSM receptor [19]. Essentially, some of the molecules mentioned above were shown to act synergistically in promoting bone destruction. For instance OSM together in combination with TNFα was demonstrated to stimulate RANK/RANKL expression in a model of inflammatory arthritis especially at the site of bone erosion [20]. Considering pro-osteoclastogenic activity of IL-6, OSM, IL-17A and TNFα, it seems reasonable to speculate that these factors (whether acting separately or simultaneously) may function as osteoclast precursors sensitizers in the course of SpA, regardless of innate monocyte functional properties in these patients.

Furthermore, intrinsic monocyte properties accompanied by dysregulated immune response may constitute an important driver of osteoclastogenesis in SpA. As described recently, exaggerated proinflammatory response may be associated with defects in autophagy in non-classical CD14^−^CD16^++^ monocytes that results in increased inflammasome activity and uncontrolled inflammatory mediators production in AS [21]. Thus, CD14^−^CD16^++^ non-classical monocytes, as one of the cellular sources of proinflammatory cytokines, would be responsible for induction of alternative osteoclastogenesis pathways and/or cytokine-producing lymphocytes that could induce osteoclast differentiation of classical CD14^++^CD16^−^ monocytes – the main source of osteoclasts in human [22]. Additionally, an upregulation of proteins involved in inflammation and the ubiquitin proteasome pathway was shown in AS monocytes [23]. Such an intrinsic property of AS monocytes could promote chronic inflammation (in line with our results showing increased levels of proinflammatory cytokines in SpA patient’s sera) that might result in (observed in our experiments) increased RANK expression and directly lead to osteoclastogenesis induction. In an arthritis mouse model, elevated RANK signalling was demonstrated to contribute to higher RANKL responsiveness and osteoclast differentiation of inflammatory non-classical monocytes [24]. In mice therefore, non-classical monocytes were shown to be pivotal cells in tissue damage in arthritis. Similar mechanism may in part play a role in human, however caution must be taken in direct transferring results obtained with mouse monocytes to human setup as monocyte populations in different species were shown not to be functionally equivalent [25]. Summaraising, the growing body of evidence suggests that in such a disease as SpA, augmented osteoclastogenesis may be secondary to dysregulated proinflammatory response.

Our outcomes should be seen in the context of their limitations. These results should be viewed as hypothesis generating. We assessed the serum levels of cytokines, and thus we do not know whether the same associations would be true for the synovial fluid, synovial and enthesial tissues and for bone tissue. What is more, all such analyses are potentially burdened with multiple-testing bias. In our comparisons of molecule levels in patients and healthy subjects, the adjustment for multiple testing, with an exception for RANKL (*P* = 0.07 from *P* = 0.016) did not alter the inferences. It seems likely that the levels of certain molecules, eg. RANKL may be higher in local microenvironment than in the serum [26]. This points out that despite of systemic low soluble RANKL concentration in axSpA patients and therefore disturbed global osteoclastogenesis, it is possible that locally produced RANKL may simultaneously induce paradoxically highly active osteoclasts from their precursors. This concept would, at least in part, explain why in axSpA patients osteogenesis is accompanied by bone loss.

## Conclusions

Our study indicates that sera from axSpA patients contain factors that promote osteoclastogenesis by up-regulating RANK mRNA level in monocytes and, by that, boosting their responsiveness to RANKL. Such mechanism would explain paradoxically higher osteoclast activity in axSpA patients compared to healthy people despite low systemic RANKL levels. As indicated by our data, these factors could include IL-6, OSM, IL-17A and TNFα, but the assessment of their functional relationship to axSpA pathophysiology requires further studies.

## Abbreviations

(rh)M-CSF: (recombinant human) monocyte colony-stimulating factor
(rh)RANKL: (recombinant human) receptor activator of nuclear factor–κB ligand
AS: ankylosing spondylitis
ASDAS: Ankylosing Spondylitis Disease Activity Score
axSpA: Axial spondyloarthritis
BASDAI: Bath Ankylosing Spondylitis Disease Activity Index
bDMARDs: Biological Disease Modifying Anti-Rheumatic Drugs
CRP: C-reactive protein
ELISA: Enzyme-linked immunosorbent assay
ESR: Erythrocyte sedimentation rate
IBP: Inflammatory back pain
IL: Interleukin
mSASSS: Modified Stokes Ankylosing Spondylitis Spinal Score
OPG: Osteoprotegerin
PBMC: Peripheral blood mononuclear cells
qPCR: Quantitative polymerase chain reaction
RANK: Receptor activator of nuclear factor–κB
sDMARDs: Synthetic Disease Modifying Anti-Rheumatic Drugs
TGFβ: Transforming growth factor beta
TNFα: Tumor necrosis factor alpha
TRAP: Tartrate-resistant acid phosphate
